# Replacing iron‐folic acid with multiple micronutrient supplements among pregnant women in Bangladesh and Burkina Faso: costs, impacts, and cost‐effectiveness

**DOI:** 10.1111/nyas.14132

**Published:** 2019-05-27

**Authors:** Reina Engle‐Stone, Sika M. Kumordzie, Laura Meinzen‐Dick, Stephen A. Vosti

**Affiliations:** ^1^ Department of Nutrition University of California – Davis Davis California; ^2^ Program in International and Community Nutrition University of California – Davis Davis California; ^3^ Department of Agricultural and Resource Economics University of California – Davis Davis California

**Keywords:** pregnancy, supplementation, multiple micronutrient supplement, cost‐effectiveness, iron‐folic acid

## Abstract

Consumption of multiple micronutrient supplements (MMS) during pregnancy offers additional benefits compared with iron‐folic acid (IFA) supplementation, but the tablets are more expensive. We estimated the effects, costs, and cost‐effectiveness of hypothetically replacing IFA supplements with MMS for 1 year in Bangladesh and Burkina Faso. Using baseline demographic characteristics from LiST and effect sizes from a meta‐analysis, we estimated the marginal effects of replacing IFA with MMS on mortality, adverse birth outcomes, and disability‐adjusted life years (DALYs) averted. We calculated the marginal tablet costs of completely replacing MMS with IFA (assuming 180 tablets per covered pregnancy). Replacing IFA with MMS could avert over 15,000 deaths and 30,000 cases of preterm birth annually in Bangladesh and over 5000 deaths and 5000 cases of preterm birth in Burkina Faso, assuming 100% coverage and adherence. We estimated the cost per death averted to be US$175–185 in Bangladesh and $112–125 in Burkina Faso. Cost per DALY averted ranged from $3 to $15, depending on the country and consideration of subgroup effects. Our estimates suggest that this policy change would cost‐effectively save lives and reduce life‐long disabilities. Improvements in program delivery and supplement adherence would be expected to improve the cost‐effectiveness of replacing IFA with MMS.

## Introduction

Adequate nutrition during pregnancy has long been recognized as essential for the health of mothers and their infants.^1^ For decades, WHO has recommended provision of iron‐folic acid (IFA) supplements as part of routine antenatal care^2^ and, as a result, most countries include IFA consumption during pregnancy in their national nutrition plan. The WHO guideline for IFA provision is based on expected effects of IFA on women's health and birth outcomes, especially low birth weight (LBW), preterm birth, maternal anemia at term, and maternal iron deficiency at term.^3^

There is ample evidence that deficiencies in other micronutrients, such as zinc, iodine, and vitamin A, affect fetal growth and development, and that these deficiencies are prevalent in low‐ and middle‐income countries.^4^ In randomized, controlled trials, multiple micronutrient supplements (MMS) during pregnancy reduced the risk of small for gestational age (SGA) and LBW, in comparison with standard IFA supplementation.^5^ Effects of MMS compared with IFA on other key outcomes, such as infant mortality, have been reported for specific subgroups.^6^ However, WHO does not currently recommend MMS for pregnant women to improve maternal and perinatal outcomes,^1^ citing gaps in evidence as well as potential increased risk of pregnancy complications owing to increased incidence of large‐for‐gestational age (LGA).

The WHO recommendation related to MMS does note that “policymakers in populations with a high prevalence of nutritional deficiencies might consider the benefits of multiple micronutrient supplements on maternal health to outweigh the disadvantages, and may choose to give multiple micronutrient supplements that include iron and folic acid.”^1^ To make this decision, policymakers must consider not only the health benefits of such a policy change, but also the costs. Moreover, they must weigh these costs against those of alternative opportunities to invest in population health, including improving the performance of current IFA programs and promoting other societal priorities.

To help guide these decisions, the objective of this analysis was to estimate the effectiveness and cost‐effectiveness of hypothetically replacing IFA supplements with MMS for 1 year in the context of an ongoing program to deliver supplements to pregnant women in Bangladesh and Burkina Faso. We first estimated the marginal effects of replacing IFA with MMS on mortality and selected birth outcomes in each context, and then summarized these effects as measured by the resulting disability‐adjusted life years (DALYs) averted. For each country, we then estimated the marginal costs of instantly and completely replacing MMS with IFA (considering costs of tablets only, but not other program transition costs, some of which occur over several years) and calculated cost per case of mortality, birth outcome, and DALY averted. We estimated all outcomes for a single year (2018) for a hypothetical scenario of 100% coverage and for current levels of IFA coverage (nationally ∼50% for Bangladesh and ∼10% for Burkina Faso), but assuming perfect program management and perfect adherence in both scenarios (i.e., no tablet waste and consumption of 180 tablets for each covered pregnancy; these assumptions will be examined in more detail in subsequent work).

## Methods

### Scenarios modeled

We calculated the additional costs and benefits of replacing all IFA tablets with MMS. For the primary analysis, we used effect sizes from all trials included in a recent meta‐analysis,^6^ using both the overall effects of MMS compared with IFA and incorporating effect modification, where statistically significant. In the primary hypothetical scenario, we assumed 100% coverage and adherence; that is, the costs and benefits were calculated on the basis of the assumption that all pregnant women received and consumed 180 tablets per pregnancy.

To examine the effect of assumptions about supplement coverage on effectiveness and cost‐effectiveness estimates, we also applied coverage estimates equivalent to current IFA coverage in Bangladesh (∼50% nationally, 60.7% urban and 47.4% rural^7^) and in Burkina Faso^a^ (10.2% nationally, 16.0% urban and 8.4% rural^8^); for these scenarios, we maintained the assumption of 180 tablets consumed per “covered” pregnancy and no tablet waste.

Finally, we also examined variation in the results when calculated using the marginal effects of MMS reported for a subset of trials that used equal doses of iron in the MMS and IFA tablets.^6^

### Model structure

All calculations were performed in MS Excel and show single‐year values for 2018. Calculations were conducted separately for four strata in each country: Urban, Male Child; Urban, Female Child; Rural, Male Child; Rural, Female Child. The strata were selected on the basis of expected differences in (1) supplementation program implementation costs, (2) program coverage, and (3) adult economic returns (urban versus rural location), (4) future labor force participation rates of children, and (5) differing marginal impact of MMS compared with IFA (male versus female children). Differences in program coverage by urban versus rural strata and effect modification by child sex are addressed in this report; remaining factors will be explored in subsequent work. The results for expected benefits (cases of mortality and birth outcomes averted, years of life lost (YLL) averted, and DALYs saved) and costs were then summed to produce national‐level estimates of benefits, costs, and cost‐effectiveness.

### Data sources and assumptions: mortality and birth outcomes

For both countries, we extracted baseline demographic information regarding the size, age structure, and urban/rural distribution of the populations from the Lives Saved Tool (LiST), using the “subnational wizard” to create separate population project files for urban and rural strata.^9^ The LiST provides estimates of the rates of stillbirths, neonatal and infant mortality, and birth outcomes (LBW, SGA, and preterm) by drawing on population estimates from UNDP and other data sources for mortality and birth outcome calculations (Table 1). To permit estimation of the marginal benefit of replacing IFA with MMS, we created separate LiST projections for both country case studies assuming either 100% IFA coverage or current IFA coverage (for Bangladesh 50.3% nationally, 60.7% urban, 47.4% rural; for Burkina Faso 10.2% nationally, 16.0% urban, 8.4% rural).^b^

**Table 1:** Population size, prevalence of effect modifiers for the marginal effect of MMS on mortality and birth outcomes, and baseline burden of natural outcomes for Bangladesh and Burkina Faso in 2018, assuming current coverage of iron‐folic acid tablets in pregnancy*a*

	Bangladesh		Burkina Faso	
	Urban	Rural	Urban	Rural
Total population,*b* *n*	66,217,830	99,776,312	6,531,635	12,838,193
Population of age 0–4 years, *n*	4,966,020	10,072,979	715,465	2,348,602
Total annual births, *n*	1,101,038	1,898,474	158,552	521,623
Male births, %	51.19	51.19	51.1	51.1
Maternal anemia, %	31.4	43.2	58.9	70.9
Maternal underweight, %	16	28	8	18
Presence of a skilled birth attendant, %	75.7	32.4	93.9	61.8
Life expectancy at birth for males,*c* years	71.8	70.6	61.3	57.2
Life expectancy at birth for females, years	75.1	74.1	62.7	58.9
Stillbirth rate,*d* deaths per 1000 live births	4.78	21.17	2.89	20.32
Early neonatal mortality rate,*e* deaths per 1000 live births*b*	10.87	21.76	10.41	19.15
Neonatal mortality rate, deaths per 1000 live births	12.94	25.91	18.75	34.47
Infant mortality rate,*f* deaths per 1000 live births	24.5	34.4	39.1	72.2
Low birth weight,*g* %	20.93	21.66	13.86	14.00
Very low birth weight,*h* %	0.42	0.43	3.91	3.95
Preterm and SGA births,*i* %	2.57	2.68	1.84	1.87
Preterm and AGA births, %	10.85	10.85	9.14	9.14
Term and SGA births, %	30.15	31.46	20.12	20.43
Term and AGA births, %	56.43	55.01	68.90	68.56

^*a*^Source: LiST model, assuming current iron‐folic acid coverage (Bangladesh: 60.7% in urban areas and 47.4% in rural areas; Burkina Faso: 16.0% urban and 8.4% rural) and 0% coverage of multiple micronutrient supplements. In the LiST projection assuming 100% coverage, birth outcome percentages are adjusted by internal algorithms to be the same for both rural and urban areas, which generate slightly different population estimates.

^*b*^Within LiST, population is estimated to be consistent with the censuses (1961, 1974, 1981, 1991, 2001, and 2011 in Bangladesh; and 1985, 1996, and 2006 in Burkina Faso) adjusted for underenumeration, and with estimates of the subsequent trends in fertility, mortality, and international migration. For Bangladesh, annual total population estimates from the Sample Vital Registration System through 2014 were also considered, as well as the 2008 voter registration for adults age 18 and over.

^*c*^For Bangladesh, life expectancy was based on life tables derived from age and sex‐specific mortality rates from the Sample Vital Registration System from 1981 up to 2015 adjusted for infant and child mortality, the 1974 Retrospective Survey of Fertility and Mortality, and the 1962/65 Population Growth Estimation Experiment. Estimates are consistent with those from the 2001 and 2010 Bangladesh Maternal Mortality Surveys (based on sibling histories and household deaths in the preceding 36 months), and data gathered from Matlab Health and Demographic Surveillance System up to 2012. For the period 1970–1975, mortality was adjusted to take into account the excess mortality associated with the 1971 civil war and independence from Pakistan, and the 1974 flood and famine. For Burkina, life expectancy was estimated using the South model of the Coale–Demeny Model Life Tables and three parameters:^1^, ^2^ direct and indirect estimates of infant and child mortality, and^3^ adjusted estimates of adult mortality (45q15). Data from West African rural demographic surveillance sites and urban vital registration were also considered. Adjusted estimates of adult mortality were derived from recent household deaths data (unadjusted and adjusted for underregistration using the growth‐balance and synthetic‐extinct generation methods) from the 1960/1961 survey, 1976, 1985, 1996, and 2006 censuses, the 1991 National Demographic Survey, and 2008 Global Fund survey; parental orphanhood from the 1993, 2003, and 2010/2011 DHS, 2006 MICS3 and 2006 census; siblings deaths from the 1998/1999, 2003, and 2010/11 DHS; intercensal survivorship from successive census age distributions (smoothed and unsmoothed) for periods 1976–1985, 1985—1996, and 1996–2006; and the implied relationship between child mortality and adult mortality based on the North model of the Coale–Demeny Model Life Tables in 1950–1955, and assumed to converge over time toward the South model of the Coale–Demeny Model Life Tables by the 1990s.

^*d*^WHO estimates for years 2000–2015 (http://datacompass.lshtm.ac.uk/115/).

^*e*^LiST does not report early neonatal mortality rates. However, Rahman Chowdhury *et al*.^20^ report that the early neonatal mortality rate in Bangladesh is 84% of the total neonatal mortality rate (https://www.ncbi.nlm.nih.gov/pmc/articles/PMC2965329/), and Koueta *et al*.^21^ report that the early neonatal deaths in Burkina Faso account for 55.6% of the total neonatal mortality.

^*f*^Infant mortality estimates are derived from the child mortality rates using the West model of the Coale–Demeny Model Life Tables and are consistent with national estimates. Child mortality estimates are based on (1) adjusted data from the Sample Vital Registration System from 1980 through 2015, (2) data on children ever‐born and surviving classified by age of mother (and the West model of the Coale–Demeny Model Life Tables), and (3) data on births and deaths under‐five calculated from maternity‐history data from the 1993/1994, 1996/1997, 1999/2000, 2004, 2007, 2011, and 2014 DHS, 2001 Bangladesh Maternal Health Services and Maternal Mortality Survey, 2009 MICS, and 2012/2013 MICS (preliminary). Levels and trends since the mid‐1980s are consistent with under‐five mortality estimates on the basis of the 2001 BMMS sibling history and data gathered from Matlab Health and Demographic Surveillance System up to 2005. In Burkina Faso, estimates of infant mortality are based on (1) data on births and deaths under‐five from maternity‐history data from the 1992/1993, 1998/1999, 2003 DHS, and 2010/11 DHS‐MICS; (2) data on recent household deaths from the 1960/1961 survey, 1976, 1985, 1996, and 2006 censuses, and the 1991 National Demographic survey; (3) data on children ever‐born and surviving classified by age of mother (and the North model of the Coale–Demeny Model Life Tables) from these data sources as well as from the 2006 MICS3 survey. Infant mortality estimates are cross‐validated and adjusted for underreporting using relationships between infant and child mortality estimates (for both sexes, and by sex) using data from 15 demographic surveillance sites and cohort studies in the Sahel region for the period 1943–1999.

^*g*^The burden of low birth weight in Burkina Faso may be biased because only approximately one third of children are weighed several days (or more) after birth (Blanc and Wardlaw^22^). However, the direction of the bias is challenging to assess for at least two reasons: heavier children are more likely to survive to be weighed, but children are known to lose body weight during the first few days after birth.

^*h*^LiST does not report very low birth weight rates. However, the National Low Birth Weight Survey of Bangladesh reports that approximately 2% of Bangladeshi infants born with low birth weight (<2500 g) have very low birth weight (<1500 g) (https://www.unicef.org/bangladesh/Low_Birth_Weight_report.pdf), and Villani *et al*.^23^ report that approximately 28.2% of Burkina Faso infants born with low birth weight have very low birth weight.

^*i*^For details, see Lee *et al*.^24^

We relied on a recent meta‐analysis by Smith *et al*. for effect sizes for the marginal impacts of MMS, compared with IFA, on mortality and birth outcomes because the analysis examined both overall effects and effects among selected subgroups, such as anemic versus nonanemia women.^6^ We focused on the following outcomes: stillbirths, mortality among live births (neonatal and infant mortality; 6‐month mortality was excluded because baseline estimates were not available from LiST), very LBW, LBW, very preterm birth, preterm birth, and small‐for‐gestational age (SGA). Following Smith *et al*.,^6^ we estimated the effects on SGA using both Oken and Intergrowth standards.^c^ We did not include estimates of the effects on LGA because baseline estimates are not available in LiST, and LGA is not considered a desirable outcome. (We note that although Smith *et al*. observed an increased risk of LGA in the multiple micronutrient group compared with the IFA group using the Intergrowth standard, but not the Oken reference, no increased risk of mortality was observed.)

A recent Cochrane review reported slightly different estimates of the overall effect of MMS compared with IFA, particularly for preterm birth and stillbirth.^10^ For this analysis, we applied overall estimates from Smith *et al*. for consistency with the modeling of subgroup effects, but we note that the overall effects on preterm birth, mortality, and DALYs would be lower if the estimates from the Cochrane review were used.

Outcomes were estimated only where the marginal effect of MMS versus IFA was statistically significant (*P* < 0.05), either for the overall analysis or for a given subgroup (in the case of statistically significant effect modification). We calculated the impacts of MMS in two ways: by applying the overall effect size to the entire population, and by incorporating effect modification (i.e., differing effects in different population subgroups) by applying subgroup‐specific effect sizes to the appropriate subset of the population, and summing these results within each stratum. For example, within each stratum, the number of cases of LBW averted by replacing IFA with MMS was calculated separately for births to women with and without anemia; results for each stratum (urban male, urban female, etc.) were then summed to calculate the total cases of LBW averted.

Smith *et al*. examined various potential effect modifiers, some of which are potentially associated with each other (e.g., maternal hemoglobin at enrollment and the presence of a skilled birth attendant (SBA)). We applied the following selection process to identify the final set of effect modifiers (population subgroups) to be included in the analysis. First, effect modifiers for which the *P* value for heterogeneity was >0.05 (on the basis of analyses in Smith *et al*.^6^) were excluded for that outcome. For example, the effect of MMS compared with IFA on preterm birth did not differ by infant sex (RR (95% CI); male 0.93 (0.88, 0.97); female 0.91 (0.86, 0.96); *P* for heterogeneity = 0.63, using data from all trials). Remaining effect modifiers were discussed on a case‐by‐case basis for each outcome, with consideration given to the (1) independence of effect modifiers, for example, the presence of an SBA and maternal adherence to supplementation may be influenced by similar factors; (2) availability of data on prevalence of effect modifiers, for example, gestational age at enrollment in the trial (initiation of supplementation) is information that would be available in a clinical trial setting but typically not in a programmatic or national distribution setting; and (3) potential for translating effect modifiers from clinical trial settings to a program setting, for example, adherence is assessed in trial settings but is not always assessed in the context of programs. Additional details on this selection process, which apply to both the Bangladesh and Burkina Faso case studies, are presented in Supplementary Tables S1 and S2 (online only).

Thus, for example, for female infants, a 14% reduction in early neonatal mortality was applied (RR = 0.86, from Smith *et al*.^6^), but *no* cases of early neonatal mortality were averted among *male* infants. For infant mortality, two effect modifiers were selected: infant sex and the presence of an SBA. In this case, we applied the following reductions in infant mortality to each subgroup: male, with SBA: 0 effect; female, with SBA: 13% reduction (RR = 0.87); males and females without SBA: 18% reduction (RR = 0.82).

We used results from all trials included in the Smith *et al*. meta‐analysis for our primary analysis. As a sensitivity analysis, we also calculated results using effect sizes from the subset of trials in which the multiple micronutrient and IFA supplements contained equal doses of iron (*n* = 8 trials). See Supplementary Tables S4 and S6 (online only) for these results for Bangladesh, and Supplementary Tables S5 and S7 (online only) for Burkina Faso.

### Data sources and assumptions: DALYs

We calculated DALYs according to the method proposed by Fox‐Rushby and Hanson^11^ and Murray and Lopez^12^ without age weighting. We generated separate estimates for YLL due to mortality (including stillbirth and postnatal mortality), disability due to LBW, and disability due to preterm birth. Because the mortality estimates included in the cost‐effectiveness analysis (neonatal, 6‐month, and infant mortality) are not independent, we calculated YLL using infant mortality rates in addition to stillbirths, which are estimated separately in LiST, and applied stratum‐specific life expectancy estimates (Table 1).

The Global Burden of Disease study reports disability weights for different consequences of preterm birth (vision, cognitive, motor, epilepsy alone or epilepsy combined with motor or cognitive impairments),^13^ but there is only one disability weight covering LBW and its consequences (from previous work^12^) and no disability weight for SGA. Therefore, for LBW, total DALYs were calculated, and for preterm birth, DALYs were estimated using several preterm birth sequelae and their estimated severities (Supplementary Table S3, online only). The same sequelae‐specific disability weights were used for the Bangladesh and the Burkina Faso case studies.

The proportion of the population affected by each condition (consequence of preterm birth) was based on estimates provided by several papers. We did not estimate YLDs for vision impairments due to preterm birth.^14^^,^ ^d^ For Bangladesh, the proportions of the population affected by each level of severity (i.e., the proportion with mild, moderate, or severe impairment) were based on estimates for severity of motor impairment by Benfer *et al*.^15^ For Burkina Faso, estimates of the same proportions were provided by Ogoke and Iloeje^16^ for the case of Nigeria, which we then assume apply to the population of preterm children in Burkina Faso.

For both case studies, we assume that the consequences of preterm birth and LBW will begin in the first year of life and persist throughout an affected individual's lifetime, and therefore applied the respective disability weight to the country‐ and stratum‐specific life expectancy of each affected individual (Table 1).

Finally, because LBW and preterm are not independent outcomes, we calculated total DALYs by combining YLL from stillbirth and infant mortality, YLDs from LBW, and YLL from stillbirth and infant mortality, and YLDs from preterm birth.

### Data sources and assumptions: tablet costs^e^

We calculated the cost of replacing IFA with MMS as the *marginal cost* of multiple micronutrient tablets compared with IFA tablets and did not consider the cost for production of capsules instead of tablets.^f^ We focus only on production costs, assuming that packaging, transportation, in‐country shipping/handling, and other costs would be very similar to those that countries are paying or would have to pay for importing and distributing IFA tablets. The micronutrient ingredient cost of a standard UNIMMAP formulation^g^ is roughly twice that of an IFA tablet containing 60 mg of iron and 400 μg of folic acid, and about three times that of an IFA tablet containing 30 mg of iron and the same amount of folic acid.^h^ We estimate that producing multiple micronutrient tablets using the UNIMMAP formula would cost approximately $US 0.004878 more than producing a tablet that contains the average amount of iron included in currently available IFA tablets^i^ and 400 μg of folic acid. This translates into a marginal increase in tablet costs of $US 4878 per million tablets. This marginal cost ignores all costs associated with, for example, changes in packaging that an IFA‐MMS switch might require. In this analysis, we are examining the benefits and costs of an immediate, complete, 1‐year switch from IFA to MMS, and therefore do not address the programmatic costs of a (likely) multiyear transition, for example, costs of meetings related to advocacy and logistics, and additional health worker training. These issues will be explored in future work.

For both country's case studies, the total number of tablets consumed was estimated by calculating the total number of annual births as the sum of live births and stillbirths (excluding miscarriages and elective terminations) and multiplying this number by the assumed number of tablets consumed per pregnancy (180) and program coverage. Tablet cost differentials are reported below in $US.

## Results

### Population characteristics and baseline burden of natural outcomes

The baseline information required to estimate the effects of shifting from IFA to MMN, for urban and rural populations in Bangladesh and Burkina Faso, appears in Table 1. Rates of stillbirth and postnatal mortality, and prevalence of birth outcomes were estimated using the LiST model; mortality was generally greater in rural areas although estimated prevalence of birth outcomes did not differ. The prevalence of population subgroups with different responses to shifting from IFA to MMS also varied between urban and rural strata. For example, in the rural stratum, the prevalence of maternal anemia and maternal underweight was greater, and the prevalence of presence of an SBA was lower.

The size and age structure of the Burkinabe population are very different from those of the Bangladeshi population; both population characteristics will affect the overall benefits and costs of shifting from IFA to MMS. Note also that child mortality rates and rates of undesirable birth outcomes vary substantially across the two case study countries. Therefore, the *composition* of the benefits of shifting from IFA to MMS is expected to vary between the two countries.

### Estimated tablet costs

In the hypothetical scenario for Bangladesh, in which all pregnant women receive and consume 180 tablets and 100% of IFA tablets are replaced with MMS, ∼550 m tablets would be consumed annually, and the additional cost to replace the IFA tablets would be $US 2.7 million (Table 2). At current coverage levels (∼50%, nationally), the cost would be $US 1.4 million, assuming 180 tablets per covered pregnancy.

**Table 2:** Estimated costs of replacing IFA tablets with multiple micronutrient tablets in Bangladesh and Burkina Faso, assuming 100% coverage, or current coverage^a^

	Bangladesh				Burkina Faso			
	100% coverage		Current coverage		100% coverage		Current coverage	
	Urban	Rural	Urban	Rural	Urban	Rural	Urban	Rural
Annual births (live births + stillbirths), *n*	1,106,845	1,938,651	1,106,305	1,938,665	159,010	532,224	159,010	532,224
Proportion of births covered, %	100	100	60.7	47.4	100	100	16.0	8.4
Number of tablets consumed per covered birth, *n*	180	180	180	180	180	180	180	180
Total number of tablets distributed annually, *n*	199,232,100	348,957,180	120,874,884	165,406,898	28,621,800	95,800,320	4,579,488	8,047,227
Total annual incremental tablet cost, USD	$971,921	$1,702,330	$589,668	$806,910	$137,262	$459,433	$21,924	$38,525

a Current coverage was estimated to be as follows: Bangladesh: 60.7% in urban areas and 47.4% in rural areas; Burkina Faso: 16.0% urban and 8.4% rural.^8^

Note: Costs are the incremental cost of the supplements ($US 0.004878 per tablet, assuming imported tablets) and exclude programmatic transition costs and tablet “waste” (i.e., in this analysis, the appropriate numbers of tablets are distributed and are consumed). We used the sum of live births and stillbirths as a proxy to estimate the annual number of pregnancies. This underestimates the number of pregnancies, since miscarriages and elective terminations of pregnancies are not included.

In Burkina Faso, if all pregnant women receive and consume 180 tablets and 100% of IFA tablets are replaced with MMS, ∼125 m tablets would be consumed annually at an additional cost of approximately $US 600,000 (Table 2). At current coverage levels (10.2% nationally, 16% in urban areas and 8.4% in rural areas), many fewer tablets (∼12.6 m) would be needed and the cost would be substantially lower (approximately $US 60,000).^j^

### Predicted effects and cost‐effectiveness for mortality and birth outcomes

Table 3 reports the predicted additional benefits in Bangladesh of replacing IFA with MMS in relation to cases of mortality averted and other adverse birth outcomes averted. These benefits are substantial for the 100% coverage scenario, but remain so even in the case of the current coverage scenario (∼50% nationally: 61% in urban areas, 47% in rural areas, WFP/UNICEF/IPHN, 2009).

**Table 3:** Marginal benefits of replacing IFA tablets with multiple micronutrient tablets for pregnant women in Bangladesh: number of cases of stillbirths, mortality, and adverse birth outcomes averted in 2018, and USD per case averted, assuming either 100% coverage or current coverage (∼50%) and using estimated overall marginal effects of MMS over IFA from all trials, and incorporating effect modification of the relationship between supplementation and the selected outcomes^a^

		Number of cases averted annually		USD per case averted	
		100% coverage	Current coverage	100% coverage	Current coverage
Stillbirths	Overall effect	3637	1780	$735.31	$784.61
	Effect modifier (n/a)	No EM	No EM	No EM	No EM
Early neonatal	Overall effect	0	0	n/a	n/a
mortality	Effect modifier (infant sex)	3587	1809	$745.44	$772.21
Neonatal mortality	Overall effect	0	0	n/a	n/a
	Effect modifier (infant sex)	4576	2307	$584.43	$605.41
Infant mortality	Overall effect	0	0	n/a	n/a
	Effect modifier (infant sex and the presence of skilled birth attendant)	11,646	5848	$229.62	$238.79
Low birth weight	Overall effect	67,807	35,452	$39.44	$39.39
	Effect modifier (maternal anemia)	68,579	37,869	$38.99	$36.88
Very low birth weight	Overall effect	2486	1300	$1075.62	$1074.37
	Effect modifier (n/a)	No EM	No EM	No EM	No EM
Very preterm birth	Overall effect	6641	3472	$402.67	$402.21
	Effect modifier (maternal underweight)	3555	1804	$752.31	$774.33
Preterm birth	Overall effect	31,438	16,437	$85.06	$84.97
	Effect modifier (maternal underweight)	33,007	17,112	$81.02	$81.62
SGA Oken	Overall effect	25,753	13,465	$103.84	$103.72
	Effect modifier (n/a)	21,541	13,710	$124.15	$101.87
SGA Intergrowth	Overall effect	42,922	22,441	$62.31	$62.23
	Effect modifier (n/a)	No EM	No EM	No EM	No EM
Total mortality (stillbirths + infant mortality)	Varied (no EM for stillbirth; infant sex and the presence of skilled birth attendant for infant mortality)	15,283	7628	$174.98	$183.08

a Results assume that each pregnant woman who is covered receives and consumes 180 capsules per pregnancy and that tablets are imported.

Note: Effect sizes for estimation of cases averted are taken from Smith *et al*.,^6^ using results from all included trials. See the text for explanation of selection of effect modifiers for each outcome.

EM, effect modification; IFA, iron‐folic acid; SGA, small for gestational age; n/a, maternal anemia.

If all pregnant women consumed the prescribed number of tablets, replacing IFA with MMS was predicted to reduce the number of stillbirths by more than 3600 cases, whereas current coverage levels could still be expected to yield 1780 cases of stillbirth averted if each covered woman took the full course of supplements. Although the overall impact of MMS compared with IFA on infant mortality was not statistically significant (Smith *et al*.^6^), a significant reduction in infant mortality would be expected to occur among *female* infants and births at which no SBA was present. Accounting for these subgroup effects, by replacing IFA with MMS, infant mortality would fall by 5848 at current coverage and by 11,646 with 100% coverage.

The predicted additional benefits for the case of Burkina Faso of replacing IFA with MMS are very different from those of Bangladesh (Table 4). Although birthrates are much higher in Burkina Faso, the population in Bangladesh is much larger than that of Burkina Faso, so all estimates of the *absolute* benefits for Burkina Faso are much smaller. In addition, as noted earlier, rates of child mortality are in general higher, and of undesired birth outcomes lower, in Burkina Faso than in Bangladesh.

**Table 4:** Marginal benefits of replacing IFA tablets with multiple micronutrient tablets for pregnant women in Burkina Faso: number of cases of stillbirths, mortality, and adverse birth outcomes averted in 2018, and USD per case averted, assuming either 100% coverage or current coverage (∼10%) and using estimated overall marginal effects of MMS over IFA from all trials, and incorporating effect modification of the relationship between supplementation and the selected outcomes^a^

		Number of cases averted annually		USD per case averted	
		100% coverage	Current coverage	100% coverage	Current coverage
Stillbirths	Overall effect	885	77	$674.45	$784.03
	Effect modifier (n/a)	No EM	No EM	No EM	No EM
Early neonatal	Overall effect	0	0	n/a	n/a
mortality	Effect modifier (infant sex)	778	74	$767.18	$819.62
Neonatal mortality	Overall effect	0	0	n/a	n/a
	Effect modifier (infant sex)	1500	142	$397.79	$424.99
Infant mortality	Overall effect	0	0	n/a	n/a
	Effect modifier (infant sex and the presence of skilled birth attendant)	4457	407	$133.88	$148.41
Low birth weight	Overall effect	10,320	1050	$57.82	$57.59
	Effect modifier (maternal anemia)	7740	1369	$50.88	$44.16
Very low birth weight	Overall effect	5336	543	$111.83	$111.38
	Effect modifier (n/a)	No EM	No EM	No EM	No EM
Very preterm birth	Overall effect	2513	240	$237.44	$251.58
	Effect Modifier (maternal underweight)	871	76	$684.73	$797.37
Preterm birth	Overall effect	5846	595	$102.07	$101.66
	Effect modifier (maternal underweight)	5520	551	$108.09	$109.69
SGA Oken	Overall effect	3903	397	$152.89	$152.28
	Effect modifier (n/a)	4825	704	$123.66	$85.88
SGA Intergrowth	Overall effect	6505	662	$91.74	$91.37
	Effect modifier (n/a)	No EM	No EM	No EM	No EM
Total mortality (stillbirths + infant mortality)	Varied (no EM for stillbirth; infant sex and the presence of skilled birth attendant for infant mortality)	5342	484	$111.71	$124.79

a Results assume that each pregnant woman who is covered receives and consumes 180 capsules per pregnancy and that tablets are imported.

Note: Effect sizes for estimation of cases averted are taken from Smith *et al*.,^6^ using results from all included trials. See the text for explanation of selection of effect modifiers for each outcome.

EM, effect modification; IFA, iron‐folic acid; SGA, small for gestational age; n/a, maternal anemia.

In Burkina Faso, replacing MMS with IFA (assuming 180 tablets per covered pregnancy) was predicted to reduce the number of stillbirths by 885 cases, whereas current coverage levels are expected to yield only 77 cases of stillbirth averted. Accounting for heterogeneity in the effects of MMS versus IFA on infant mortality, replacing IFA with MMS would reduce infant mortality by 407 cases in 2018 at current coverage and by 4457 with 100% coverage.

Accounting for effect modification only slightly modified the number of cases of preterm and SGA avoided. However, the number of cases of SGA avoided was almost twice as high when using effect sizes for the Intergrowth standard compared with the Oken reference (assuming 100% coverage, Bangladesh: 42,922 Intergrowth versus 25,753 Oken; Burkina Faso: 6505 Intergrowth versus 3903 Oken).

For both countries, the number of cases of mortality or adverse birth outcomes averted in the current coverage scenario was approximately proportional to the number of cases averted in the 100% coverage scenario (i.e., approximately half in Bangladesh and ∼10% in Burkina Faso). However (predictably, in this analysis), the cost per case averted was very similar since only the marginal cost of tablets was included and we assumed 100% adherence among covered pregnancies and no waste in the distribution of tablets.

In Bangladesh (Table 3), the predicted cost per death averted ranged from ∼US$239 (infant mortality, with effect modification) to ∼US$785 (stillbirths, overall effect), or ∼US$184 per death averted in total (stillbirths + infant mortality); equivalent values for Burkina Faso (Table 4) ranged from ∼US$148 (infant mortality, with effect modification) to ∼US$784 (stillbirths, overall effect) and ∼US$125 per death averted in total. For adverse birth outcomes, the cost per case averted in Bangladesh ranged from ∼US$37 per case of LBW averted (with effect modification) to ∼US$1074 per case of very LBW averted (overall effect). In Burkina Faso, the cost per case averted ranged from ∼US$44 per case of LBW averted (with effect modification) to ∼US$797 per case of very preterm birth averted (overall effect).

### Predicted effects and cost‐effectiveness of shifting from IFA to MMS for YLL, YLD, and DALYs averted

Predicted numbers of YLL, years lived with disability (YLD), and DALYs averted by shifting from IFA to MMS for Bangladesh and Burkina Faso are presented in Tables 5 and 6, respectively, using overall effects and accounting for effect modification (subgroup effects), for both the 100% coverage and for the current coverage scenarios. In Bangladesh, estimated effects for the ∼50% current coverage scenario were approximately half those estimated in the hypothetical 100% coverage scenario; total predicted DALYs averted at 100% coverage ranged from ∼209,200 (DALYs estimated using YLL + preterm YLD, overall effect of MMS) to ∼747,500 (DALYs estimated using YLL + LBW YLD, with effect modification). YLL constituted 34% and 57% of total DALYs estimated using LBW and preterm, respectively, when overall effects were applied, and 68% and 84% of total DALYs when effect modification was taken into account. The predicted cost per YLL, YLD, and DALY averted was almost identical between the two coverage scenarios, with the cost per DALY averted ∼US$7–13 for overall effects and US$3–5 when accounting for effect modification.

**Table 5:** Marginal benefits of replacing IFA tablets with multiple micronutrient tablets for pregnant women in Bangladesh: disability‐adjusted life‐years (DALYs), and USD per DALY averted in 2018, assuming either 100% coverage or current coverage (∼50%) and using estimated overall marginal effects of MMS over IFA from all trials and incorporating effect modification of the relationship between supplementation and the selected outcomes^a^

		Number of YLL, YLD, or DALYs averted		USD per YLL, YLD, or DALY averted	
		100% coverage	Current coverage	100% coverage	Current coverage
YLL (mortality)	Overall effect	118,824	58,142	$22.51	$24.02
	Effect modifier (multiple)	510,386	254,708	$5.24	$5.48
YLD (low birth weight)	Overall effect	234,378	122,486	$11.41	$11.40
	Effect modifier (maternal anemia)	237,083	130,861	$11.28	$10.67
YLD (preterm birth)	Overall effect	90,414	47,250	$29.58	$29.56
	Effect modifier (maternal underweight)	94,653	49,067	$28.25	$28.46
Total (mortality + LBW)	Overall effect	353,202	180,628	$7.57	$7.73
	Effect modifier (multiple)	747,469	385,569	$3.58	$3.62
Total (mortality + preterm)	Overall effect	209,238	105,392	$12.78	$13.25
	Effect modifier (multiple)	605,036	303,775	$4.42	$4.60

a Results assume that each pregnant woman who is covered receives and consumes 180 capsules per pregnancy and that tablets are imported.

Note: Effect sizes for estimation of cases averted are taken from Smith *et al*.,^6^ using results from all included trials. See the text for explanation of selection of effect modifiers for each outcome.

DALY, disability‐adjusted life year; YLD, years lived with disability; YLL, years of life lost.

**Table 6:** Marginal benefits of replacing IFA tablets with multiple micronutrient capsules for pregnant women in Burkina Faso: disability‐adjusted life‐years (DALYs), and USD per DALY averted in 2018, assuming either 100% coverage or current coverage (∼10%) and using estimated overall marginal effects of MMS over IFA from all trials and incorporating effect modification of the relationship between supplementation and the selected outcomes^a^

		Number of YLL, YLD, or DALYs averted		USD per YLL, YLD, or DALY averted	
		100% coverage	Current coverage	100% coverage	Current coverage
YLL (mortality)	Overall effect	27,697	2412	$21.54	$25.06
	Effect modifier (multiple)	171,190	15,510	$3.49	$3.90
YLD (low birth weight)	Overall effect	34,197	3474	$17.45	$17.40
	Effect modifier (maternal anemia)	38,865	4531	$15.35	$13.34
YLD (preterm birth)	Overall effect	15,372	1562	$38.82	$38.71
	Effect modifier (maternal underweight)	14,566	1454	$40.97	$41.58
Total (mortality + LBW)	Overall effect	61,894	5887	$9.64	$10.27
	Effect modifier (multiple)	210,055	20,042	$2.84	$3.02
Total (mortality + preterm)	Overall effect	43,069	3974	$13.85	$15.21
	Effect modifier (multiple)	185,756	16,964	$3.21	$3.56

a Results assume that each pregnant woman who is covered receives and consumes 180 capsules per pregnancy and that tablets are imported.

Note: Effect sizes for estimation of cases averted are taken from Smith *et al.*,^6^ using results from all included trials. See the text for explanation of selection of effect modifiers for each outcome.

YLD, years lived with disability; YLL, years of life lost.

In Burkina Faso (Table 6), for all summary benefit measures, current (∼10%) coverage rates of IFA (and by assumption, multiple micronutrient) programs are very low relative to 100% coverage estimates, but again, cost‐effectiveness estimates were roughly similar because of the single‐year, tab‐cost‐only focus of this analysis. That said, under current coverage, the cost per DALY averted by shifting from IFA to MMS in the context of Burkina Faso was similar to that for Bangladesh, ranging from approximately US$3 to US$15.

### Sensitivity analyses

Results for mortality and birth outcomes, and for summary YLL, YLD, and DALY averted, were similar when effect sizes from the meta‐analysis limited to trials in which *equal doses of iron* were used in the IFA and multiple micronutrient groups were applied (Supplementary Tables S4 and S6, online only, for the case of Bangladesh, and Supplementary Tables S5 and S7, online only, for Burkina Faso). Sensitivity analyses of the cost per YLL and total DALYs averted associated with changes in assumed tablet costs are presented in Supplementary Table S8 (online only) for Bangladesh and in Supplementary Table S9 (online only) for Burkina Faso. Under the assumptions of this analysis (180 tablets per covered pregnancy), changes in the cost per YLL and DALY averted were proportional to changes in the incremental tablet cost (i.e., doubling the incremental cost per tablet would double the cost per DALY averted).

## Discussion

Policymakers in LMICs are aware that women of reproductive age in general, and pregnant women in particular, are lacking in some of the micronutrients that are essential for healthy pregnancies and healthy infants. One method for addressing these micronutrient deficiencies is the shift from the decades‐long practice of providing IFA to pregnant women to providing multiple micronutrient tablets that contain iron, folic acid, and an array of other micronutrients. In this study, we developed a model to provide estimates of the expected benefits and the expected costs of such a transition in the very different contexts of Bangladesh and Burkina Faso, if done immediately in both cases for 1 year, and if the only additional cost were that associated with purchasing the more expensive multiple micronutrient tablets.

The expected benefits of the shift from IFA to MMS are substantial, both with reference to reductions of mortality and undesirable birth outcomes (e.g., LBW). Under the assumption that all pregnant women are treated with a regimen of 180 tablets consumed over their pregnancies, shifting from IFA to MMS was predicted to save over 15,000 young lives in 1 year in Bangladesh (combining stillbirths and infant mortality) and approximately 30,000 cases of preterm birth would be averted. In all, ∼600,000 DALYs would be averted from a 1‐year switch. Most of these measures of impact are reduced by approximately one‐half if current coverage of the IFA program (∼50% nationally) is imposed in the model. Nonetheless, even at current coverage levels, the savings in lives and in YLDs are expected to be substantial in Bangladesh if adherence to the prescribed number of tablets is high.

The expected benefits of shifting from IFA to MMS in the context of Burkina Faso are much smaller, in part because the overall population is smaller but also because the current coverage of the IFA program is low (∼10% nationally).^8^ Under the current‐coverage scenario, the model predicts that a 1‐year shift from IFA to multiple micronutrient tablets would save 484 young lives (stillbirth and infant mortality) and eliminate over 550 cases of preterm birth in that year, which would result in ∼17,000 DALYs being avoided. Because current coverage in Burkina Faso was estimated from DHS data as the share of women who reported that they took IFA supplements for 180 or more days during their previous pregnancy, these are likely conservative estimates of the benefits of replacing IFA with MMS in Burkina Faso. That is, approximately 40% of women in Burkina Faso reported consuming at least 90 but fewer than 180 IFA tablets, and any benefits (and costs) of this level of consumption were not captured in this analysis. On the other hand, the results for Bangladesh may overestimate the benefits of this switch since the available definition of coverage did not specify a minimum number of tablets consumed. However, it is important to note that the *cost‐effectiveness* of the outcomes examined did not vary appreciably by coverage under this scenario. That is, the absolute benefits of this programmatic change would increase with increasing coverage, but the relative value of investing in replacing IFA with MMS compared with other health investments does not depend on coverage (under a scenario in which the only costs are those of the tablets themselves and all covered pregnancies are assumed to receive and consume 180 tablets; as noted elsewhere, the cost‐effectiveness of varying numbers of tablets distributed and consumed will be examined in subsequent work).

The costs associated with shifting from IFA to MMS will be significant, mainly because the multiple micronutrient tablets are approximately 35% more expensive than IFA tablets and, in the context of Bangladesh, hundreds of millions of tablets would be required each year. In sum, the complete and immediate shift from IFA to MMS for 1 year in Bangladesh, given current coverage levels, would be approximately US$1.7 million, but costs would rise to US$2.7 million if sufficient tablets were purchased to cover *all* pregnancies with 180 tablets per person. In Burkina Faso, making the IFA‐MMS shift only for those currently receiving the full regimen of tablets (at least 180 per person) would require only approximately 12.5 m multiple micronutrient tablets at an estimated cost of approximately US$ 60,000; accounting for tablets distributed to women who consumed less than 180 tablets during pregnancy would increase these costs.

The total (tablet) cost of shifting from IFA to multiple micronutrient tablets will depend on the negotiated prices of both types of tablets, which will depend heavily on the volume and continuity of purchasing agreements, which will simultaneously involve discussions with tablet ingredient suppliers regarding the prices of the micronutrients needed to produce them. For example, Vitamin Angels, one of the largest international purchasers of multiple micronutrient tablets, paid ∼0.010722 US$ per tablet (delivered, in 180‐tablet containers, to their U.S.‐based warehouses) in 2018. This is a lower per‐tablet cost than suggested by others (e.g., 0.0111 US$ per tablet, including packaging and USP certification, but not shipping).^k^ Scale economies in tablet production are very substantial and developing countries may not have sufficient demand or negotiating leverage to secure the lowest possible tablet costs. The cost‐effectiveness of shifting from IFA to MMS will depend, in part, on the cost of multiple micronutrient tablets, which will be negotiated outcomes. The sensitivity analyses presented here explore the implications for cost‐effectiveness of these outcomes. For example, a 50% reduction in the marginal tablet cost would increase the cost‐effectiveness from ∼$3 to 15/DALY averted across the two countries to ∼$2 to 8/DALY averted, whereas a 50% increase in the marginal tablet cost would reduce the cost‐effectiveness to ∼$6 to 30/DALY averted.

Cost‐effectiveness of this narrow, 1‐year programmatic experiment suggests that this policy change is worthy of consideration.^l^ For example, the cost per case of total mortality averted (considering both stillbirths and infant mortality, and accounting for subgroup effects for the latter) under current‐coverage scenarios in each country is approximately US$183 in Bangladesh and US$125 in Burkina Faso. The cost‐effectiveness of the IFA‐MMS shift for averting child mortality is generally favorable when compared with other mortality‐averting policy options: for example, pneumococcus and rotavirus vaccines in low‐income countries tended to be more cost‐effective (from less than US$100/death averted), while most other life‐saving interventions tended to be much less cost‐effective (e.g., for rotavirus and HiB vaccines in lower‐middle income countries, over $1000 per death averted).^17^ In addition, training traditional birth attendants to reduce mortality due to birth asphyxia, hypothermia, and neonatal sepsis in Zambia was estimated to cost $591 per death averted in the optimistic scenario and $1866 per death averted in the “base case” scenario,^18^ and a package of interventions including home visits for early neonatal care in Bangladesh was estimated to cost $2939 per death averted.^19^ The cost‐effectiveness is also greater than that estimated for other interventions targeted to pregnant women. For example, women's groups for prenatal and newborn care were estimated to cost more than $1000 per death averted.

Similarly, since most of the lives saved by shifting from IFA to MMS would occur very early in life, by the summary measure of benefits, DALYs, this policy action compares very favorably with most alternatives in Bangladesh and Burkina Faso. In current‐coverage scenarios, the cost per DALY averted by shifting from IFA to MMS ranges from US$3.62 to US$13.25 in Bangladesh, and from US$3.02 to US$15.21 in Burkina Faso, depending on the method used. These cost‐effectiveness ranges are comparable to those of other interventions that save lives and reduce disability burdens, for example, zinc plus oral rehydration (cost‐effectiveness range ∼US$10–US$80 per DALY averted).^17^

The analyses underlying the estimated benefits and costs associated with shifting from IFA to MMS presented here have several important limitations. First, the analysis assumes a complete, immediate, and costless shift from IFA to MMN. This is unlikely to be the case in any country and future work will focus on the consequences for benefits and costs of relaxing these assumptions. More specifically, all transitions from IFA to MMS will take time and require investments—all of these costs will be faced up‐front and will erode the cost‐effectiveness of shifting from IFA to multiple micronutrient tablets. That said, in countries where IFA programs are functioning well (i.e., with high coverage rates), we expect the bulk of the cost increases to reflect the more expensive multiple micronutrient tablets. However, in countries with low‐performing IFA programs, the benefits associated with shifting from IFA to multiple micronutrient tablets could be attenuated by program transition costs.

Second, the analysis assumes that all “covered” pregnant women receive and consume exactly 180 tablets, a threshold that is generally consistent with the doses given in the trials that generated the measures of effect used in this analysis. It is reasonable to expect that there may be benefits associated with consumption of a smaller number of tablets (e.g., 150 instead of 180), but existing data are insufficient to construct a dose–response curve for number of tablets consumed and each of the outcomes measured here. Thus, it was necessary to select a threshold to apply the expected benefits of supplementation, recognizing that this approach may underestimate the benefits of multiple micronutrient delivery.

Third, this analysis ignores other potential benefits of multiple micronutrient provision for the mother and child, such as correcting maternal deficiencies in nutrients such as zinc or thiamin. Instead, we relied on a recent meta‐analysis for estimates of the benefits of MMS versus IFA with regard to mortality and adverse birth outcomes. Our approach takes advantages of the rigorous evidence base for these selected outcomes but may underestimate the overall benefits of replacing IFA with MMS.

Finally, consumption by all covered women of an exact dose of tablets will never be the case programmatically—some women will consume fewer than 180 tablets and others may consume more than 180 tablets—in the current model, in *both* cases, the costs of an IFA‐to‐multiple micronutrient shift will increase, but the expected benefits will not.^m^ In addition, tablet waste associated with programmatic inefficiencies (e.g., tablets not consumed before their expiration dates) was not addressed in this study—these costs would be greater for the more expensive MMS than for IFA, *ceteris paribus*. Future work will address these issues, too.

The study has several major strengths. First, we develop and use the first model to explicitly and transparently estimate the benefits and the costs associated with shifting from IFA to MMS for a disaggregated population, including varying population characteristics (baseline rates of birth outcomes) and prevalence of characteristics that modify the effects of MMS compared with IFA. Second, the model can compare benefits and costs, and hence generate estimates of cost‐effectiveness for specific mortality and adverse birth outcomes, and summary measures (e.g., DALYs). Third, the key underlying assumptions regarding population characteristics, coverage rates of IFA programs, and tablet costs can be modified easily in the model to assess the effects of changes in parameters on the effects, costs, and cost‐effectiveness of shifting from IFA to MMS. Fourth, and related, the model can easily be applied to different countries, or regions within countries.

The results presented in this paper will be of immediate interest to policymakers in Bangladesh and Burkina Faso, and in countries with similar characteristics, particularly with regard to the burden of adverse birth outcomes. The results of this simple application of the model suggest that shifting from IFA to MMS likely will be very cost‐effective. More complete and realistic scenarios will likely reduce estimates of cost‐effectiveness, but transition costs alone are not likely to reduce estimates to levels below those that merit policy attention in countries with reasonably well‐performing IFA programs. The same may not be true for countries with low antenatal care attendance or poor‐performing IFA programs. Adherence to consumption protocol (180 tablets per pregnancy), and the potential for consumption of multiple micronutrient tablets below or in excess of the 180‐tablet protocol may, on the other hand, dramatically reduce expected benefits of shifting from IFA to MMS, so policy and programmatic attention should focus on this key issue.

Policymakers in other countries should consider adopting and modifying the underlying model to better fit their realities (demographic characteristics, micronutrient deficiencies, IFA program performance, etc.) and use it to better inform the policy discussions around the shift from IFA to MMS. The results presented here will provide insights into key country‐specific characteristics that are likely to lead to higher (or lower) expected benefits, and to higher (or lower) expected costs.

## Conclusions

Population‐based models should be developed and used to predict the mortality and birth outcome benefits, and the costs, of shifting from IFA to MMS during pregnancy. A model developed for the cases of Bangladesh and Burkina Faso and used to explore the single‐year benefits and costs of a complete and immediate shift from IFA to MMS suggests that this policy would cost‐effectively save lives and reduce life‐long disabilities. Further research is needed on more realistic transition scenarios for shifting from IFA to MMS, on more complete characterization of delivery platform performance, and on more complete modeling of adherence by pregnant women to prescribed supplementation regimens. Stronger economic cases can be made for switching from IFA to MMS as the threshold number of tablets required to accrue benefits declines, and improvements in program delivery and supplement adherence would be expected to improve the cost‐effectiveness of replacing IFA with MMS.

## Competing interests

The authors declare no competing interests.

## Supporting information


**Supplementary Table S1**. Selection of effect modifiers for analyses based on *all‐trials* results for case studies in Bangladesh and Burkina Faso*^a^*

**Supplementary Table S2**. Selection of effect modifiers for analyses based on trials with *equal doses of iron* (60 mg) for case studies in Bangladesh and Burkina Faso*^a^*

**Supplementary Table S3**. Assumptions used to calculate years of life lost and disability‐adjusted life years averted: Disability weights and severity of impairment for case studies in Bangladesh and Burkina Faso

**Supplementary Table S4**. Marginal benefits of replacing iron‐folic acid tablets with multiple micronutrient tablets for pregnant women in Bangladesh based on equal‐dose iron trials: Number of cases of stillbirths, mortality, and adverse birth outcomes averted in 2018, and USD per case averted, assuming 100% coverage and current coverage (∼50%), and estimated using overall marginal effects of MMS over IFA from *equal‐dose iron trials* (unless otherwise indicated) and incorporating effect modification of the relationship between supplementation and the selected outcomes*^a^*

**Supplementary Table S5**. Marginal benefits of replacing iron‐folic acid tablets with multiple micronutrient tablets for pregnant women in Burkina Faso based on equal‐dose iron trials: Number of cases of stillbirths, mortality, and adverse birth outcomes averted in 2018, and USD per case averted, assuming 100% coverage and current coverage (∼10%), and estimated using overall marginal effects of MMS over IFA from *equal‐dose iron trials* (unless otherwise indicated) and incorporating effect modification of the relationship between supplementation and the selected outcomes*^a^*

**Supplementary Table S6**. Marginal benefits of replacing iron‐folic acid tablets with multiple micronutrient capsules for pregnant women in Bangladesh based on equal‐dose iron trials and all trials: Disability‐Adjusted‐Life Years (DALYs) in 2018 and USD per DALY averted, assuming either 100% coverage and current coverage and estimated using overall marginal effects of MMS over IFA from *equal‐dose iron trials* (unless otherwise indicated) and incorporating effect modification of the relationship between supplementation and the selected outcomes*^a^*

**Supplementary Table S7**. Marginal benefits of replacing iron‐folic acid tablets with multiple micronutrient capsules for pregnant women in Burkina Faso based on equal‐dose iron trials and all trials: Disability‐Adjusted‐Life Years (DALYs) in 2018 and USD per DALY averted, assuming either 100% coverage and current coverage (∼10%) and estimated using overall marginal effects of MMS over IFA from *equal‐dose iron trials* (unless otherwise indicated) and incorporating effect modification of the relationship between supplementation and the selected outcomes*^a^*

**Supplementary Table S8**. Sensitivity of cost‐effectiveness of replacing iron‐folic acid tablets with multiple micronutrient tablets for pregnant women in Bangladesh: USD per DALY averted under low, best‐guess, and high marginal costs of multiple micronutrient tablets, assuming current national coverage (∼50%) and estimated using overall marginal effects of MMS over IFA from all trials and incorporating effect modification of the relationship between supplementation and the selected outcomes*^a^*

**Supplementary Table S9**. Sensitivity of cost‐effectiveness of replacing iron‐folic acid tablets with multiple micronutrient tablets for pregnant women in Burkina Faso: USD per DALY averted under low, best‐guess, and high marginal costs of multiple micronutrient tablets, assuming current national coverage (∼10%) and estimated using overall marginal effects of MMS over IFA from all trials and incorporating effect modification of the relationship between supplementation and the selected outcomes*^a^*

## Footnotes

1 Analysis of Demographic and Health Surveys (DHS) microdata allowed for a more refined assessment of IFA consumption in Burkina Faso: nationally 51.4% (59.2% in urban areas, 48.91% in rural areas) of pregnant women reported consuming at least 90 IFA tablets, and 10.2% (16.0% urban and 8.4% rural) reported consuming at least 180 IFA tablets during their most recent pregnancy. Similar data are not available for IFA consumption in Bangladesh, for which the coverage estimates applied here are the proportion of women who took any IFA during their previous or current pregnancy (WFP/UNICEF/IHPN 2009). The results presented here assume that all covered pregnant women consume exactly 180 tablets, which is consistent with the coverage calculated for Burkina Faso but likely an overestimate of the benefits in Bangladesh.

2 LiST projections were generated using version 5.63 of LiST, which includes an effect of IFA on SGA. An updated version of the tool (version 5.71) does not include an effect of IFA on birth outcomes. All future simulations will be run in SAS and using the results of Spectrum version 5.7; cross‐model comparisons confirm that *all* differences in the results generated by the Microsoft Excel and SAS models are driven exclusively by the different versions of the Spectrum software on which the models depend.

3 LiST uses the Alexander standard to define SGA.

4 Blencowe *et al*.^14^ estimate the share of the population affected on the basis of the presence of neonatal intensive care units (NICU) because rates of retinopathy of prematurity (ROP) are dependent on the quality of care received. Access to NICU in Bangladesh and Burkina Faso is low, and the share of the population affected by ROP is estimated to be very small.

5 Very few low‐income countries have the national capacity to produce the quantities or qualities of multiple micronutrient tablets that would be required to replace IFA tablets. Therefore, in this analysis, all costs are based on tablets produced in Europe.

6 This analysis focuses exclusively on IFA and multiple micronutrient *tablets*. These micronutrients can also be delivered in the form of capsules, which are expected to cost approximately 10% more than tablets (K. Kramer, 2018. Personal communication).

7 The commonly used UNIMMAP formulation includes the following micronutrients: iron (mg) 30; folic acid (μg) 400; Vit A (μg) 800; Vit D (IU) 200; Vit E (mg) 10; Vit C (mg) 70; Vit B1 (mg) 1.4; Vit B2 (mg) 1.4; niacin (mg) 18; Vit B6 (mg) 1.9; Vit B12 (μg) 2.6; zinc (mg) 15; copper (mg) 2; selenium (μg) 65; iodine (μg) 150 (UNICEF/WHO/UNU 1999).

8 K. Kramer, 2018. Personal communication.

9 There is some uncertainty around the quantity of iron that will be included in multiple micronutrient tablets. For cost analyses presented here, we used an average of the amounts of iron ((60 mg + 30 mg)/2 = 45 mg) in commonly available IFA tablets.

10 The roughly linear relationship between coverage and the tablet needs and costs of shifting from IFA to MMS in the context of this single‐year, immediate‐switch experiment is to be expected—low‐coverage IFA programs are less expensive to convert to MMS because fewer tablets are distributed. However, the role of program performance in determining the cost‐effectiveness of making the shift from IFA to MMS will become apparent when the (1) timeframe for analysis is extended, (2) transition costs are incorporated, and, in particular, (3) marginal costs of tablets consumed below and in excess of the prescribed 180‐tablet protocol are included. We will address all of these issues in future work.

11 K. Kramer, 2018. Personal communication.

12 That said, preliminary analyses involving programmatic transition costs and inefficiencies, and especially of adherence by women to the suggested IFA (and by assumption, multiple micronutrient) protocol, on cost‐effectiveness estimates suggest that under some sets of assumptions, costs per outcome or DALY averted could increase by fourfold.

13 A dose–response relationship between number of tablets consumed and expected health outcomes could be postulated, but data are insufficient to model potential increases in effects beyond 180 tablets or smaller but nonzero effects among individuals who consumed some, but fewer than 180, tablets.
