# Supplementary material for: Replacing iron‐folic acid with multiple micronutrient supplements among pregnant women in Bangladesh and Burkina Faso: costs, impacts, and cost‐effectiveness
Source: Ann N Y Acad Sci. 2019 May 27;1444(1):35–51. doi: 10.1111/nyas.14132 (PMC6771790; doi:10.1111/nyas.14132)
Supplement: Supplementary file 1 — Supplementary Table S1. Selection of effect modifiers for analyses based on all‐trials results for case studies in Bangladesh and Burkina Fasoa [file NYAS-1444-35-s001.docx]

**Supplemental Table 1:** Selection of effect modifiers for analyses based on *all-trials* results for case studies in Bangladesh and Burkina Faso^1^

| **Outcomes** | **Significant effect modifiers selected** | **Significant effect modifiers not selected and reason for decision** |
| --- | --- | --- |
| Stillbirth | N/A | Gestational age at randomization  Reason: Challenges with obtaining this data in a non-trial setting. |
| Early neonatal mortality | Infant sex | Maternal education, Adherence  Reason: No significant effect within the strata although there was overall significant interaction. |
| Neonatal mortality | Infant sex | Maternal education, Adherence  Reason: No significant effect within the education strata although there was overall significant interaction. Coverage data in DHS could be used as a proxy for adherence but difficult to interpret. |
| 6-month mortality | Maternal anemia at enrollment | Presence of skilled birth attendant  Reason: Both factors are likely associated; measures of maternal anemia more standardized and more likely to be available |
| Infant mortality | Infant sex and Presence of skilled birth attendant | Adherence  Reason: Adherence likely associated with presence of skilled birth attendant. Coverage data in DHS could be used as a proxy but difficult to interpret |
| Very low birth weight | N/A | N/A |
| Low birth weight | Maternal anemia at enrollment | N/A |
| Very preterm birth | Maternal underweight at enrollment | N/A |
| Preterm | Maternal underweight at enrollment | Gestational age at randomization, Maternal anaemia at enrollment  Reason: Gestational age at enrollment likely associated with selected effected modifiers. Challenges with obtaining this data in a non-trial setting. |
| Small-for-gestational age (Oken) | Maternal anemia at enrollment | Gestational age at randomization, Maternal education  Reason: Maternal education and gestational age at randomization likely associated with selected effect modifier. Challenges with obtaining this data in a non-trial setting. |
| Small-for-gestational age (Intergrowth) | N/A | N/A |

^1^ Selection process was based on criteria set by the research team and on what is known about the availability of data on each effect modifier.
