# Supplementary material for: Replacing iron‐folic acid with multiple micronutrient supplements among pregnant women in Bangladesh and Burkina Faso: costs, impacts, and cost‐effectiveness
Source: Ann N Y Acad Sci. 2019 May 27;1444(1):35–51. doi: 10.1111/nyas.14132 (PMC6771790; doi:10.1111/nyas.14132)
Supplement: Supplementary file 2 — Supplementary Table S2. Selection of effect modifiers for analyses based on trials with equal doses of iron (60 mg) for case studies in Bangladesh and Burkina Fasoa [file NYAS-1444-35-s002.docx]

**Supplemental Table 2:** Selection of effect modifiers for analyses based on trials with *equal doses of iron* (60 mg) for case studies in Bangladesh and Burkina Faso^1^

| Outcomes | Significant effect modifiers selected | Significant effect modifiers not selected |
| --- | --- | --- |
| Stillbirth | N/A | N/A |
| Early neonatal mortality | Infant sex | N/A |
| Neonatal mortality | Infant sex | N/A |
| 6-month mortality | Maternal anemia at enrollment | Presence of skilled birth attendant  Reason: Both factors are likely associated; measures of maternal anemia more standardized and more likely to be available |
| Infant mortality | Infant sex | Adherence  Reason: Coverage data in DHS could be used as a proxy but difficult to interpret |
| Low birth weight | Maternal anemia at enrollment | Maternal education  Reason: Maternal education likely associated with selected effect modifier. Challenges with obtaining this data in a non-trial setting. |
| Preterm | Maternal underweight at enrollment | Gestational age at randomization  Reason: Likely associated with selected effected modifier. Challenges with obtaining this data in a non-trial setting. |
| Small-for-gestational age (Oken)^2^ | Maternal anemia at enrollment | Gestational age at randomization  Reason: Likely associated with selected effected modifier. Challenges with obtaining this data in a non-trial setting. |

^1^Data on effect modifiers for very low birth weight and very preterm birth were not provided in the equal-dose trial results reported in Smith et al. (2017)

^2^Data on effect modifiers were not provided for the Intergrowth standard (both SGA and LGA) in the equal-dose trial results reported in Smith et al. (2017)
