# Supplementary material for: Replacing iron‐folic acid with multiple micronutrient supplements among pregnant women in Bangladesh and Burkina Faso: costs, impacts, and cost‐effectiveness
Source: Ann N Y Acad Sci. 2019 May 27;1444(1):35–51. doi: 10.1111/nyas.14132 (PMC6771790; doi:10.1111/nyas.14132)
Supplement: Supplementary file 3 — Supplementary Table S3. Assumptions used to calculate years of life lost and disability‐adjusted life years averted: Disability weights and severity of impairment for case studies in Bangladesh and Burkina Faso [file NYAS-1444-35-s003.docx]

**Supplemental Table 3.** Assumptions used to calculate years of life lost and disability-adjusted life years averted: Disability weights and severity of impairment for case studies in Bangladesh and Burkina Faso

|  | **Severity of Impairment** | **Disability weight^4^** | **Prevalence of condition in preterm births^5^ in Bangladesh** | **Prevalence of condition in preterm births in Burkina Faso** |
| --- | --- | --- | --- | --- |
| Low birth weight^1^ |  | 0.106 | n/a | n/a |
| Preterm^2^: Motor impairment^3, 4^ | None | 0 | 0.63 | 0.63 |
|  | Mild | 0.01 | 0.086 | 0.1739 |
|  | Moderate | 0.061 | 0.114 | 0.0259 |
|  | Severe | 0.402 | 0.169 | 0.1702 |
| Preterm: Cognitive impairment |  | 0.021 | 0.5^6^ | 0.^6^ |

^1^Disability weight for low birth weight, all sequela, from GBD 1990 (1)

^2^We assume that preterm birth prevalence is 13.5% in Bangladesh and 11.0% in Burkina Faso (LiST).

^3^For the distribution of motor impairments among preterm births see Spittle et al. (2018) (2) for the prevalence of no impairment for both countries, and Benfer et al. (2014) (3) and for Bangladesh and Ogoke and Iloeje (2017) (4) for the effect for Nigeria which was applied to Burkina Faso

^4^The effects of disabilities are assumed to begin in the first year of life and to persist throughout the lifetimes of affected individuals. See Table 1 for stratum-specific life expectancy estimates.

^5^Ideally, break-down by gestational age at birth would be presented a more refined way (e.g., as reported in Shah et al. 2014 (5)); the model can be modified to do so, but supporting data are generally lacking.

^6^Authors’ assumption.

1. Murray C, Lopez A, World Health Organization, World Bank, Health HSoP. The global burden of disease: a comprehensive assessment of mortality and disability from diseases, injuries, and risk factors in 1990 and projected to 2020: summary. Geneva: World Health Organization; 1996.

2. Spittle A, Cameron K, Doyle L, Cheong J. Motor impairment trends in extremely preterm children: 1991-2005. Pediatrics. 2018;141(4).

3. Benfer K, R J, Bandaranayake S, Finn C, Ware R, Boyd R. Motor severity in children with cerebral palsy studied in a high-resource and low-resource country. Pediatrics. 2014;134(6):e1594-e602.

4. Ogoke C, Iloeje S. Severity of motor dysfunction in children with cerebral alsy seen in Enugu, Nigeria. The Pan African Medical Journal. 2017;27.

5. Shah R, Mullany L, Darmstadt G, Mannan I, Rahman S, Talukder R, et al. Incidence and risk factors of preterm birth in a rural Bangladeshi cohort. BMC Pediatr. 2014;14.
