# Supplementary material for: Replacing iron‐folic acid with multiple micronutrient supplements among pregnant women in Bangladesh and Burkina Faso: costs, impacts, and cost‐effectiveness
Source: Ann N Y Acad Sci. 2019 May 27;1444(1):35–51. doi: 10.1111/nyas.14132 (PMC6771790; doi:10.1111/nyas.14132)
Supplement: Supplementary file 5 — Supplementary Table S5. Marginal benefits of replacing iron‐folic acid tablets with multiple micronutrient tablets for pregnant women in Burkina Faso based on equal‐dose iron trials: Number of cases of stillbirths, mortality, and adverse birth outcomes averted in 2018, and USD per case averted, assuming 100% coverage and current coverage (∼10%), and estimated using overall marginal effects of MMS over IFA from equal‐dose iron trials (unless otherwise indicated) and incorporating effect modification of the relationship between supplementation and the selected outcomesa [file NYAS-1444-35-s005.docx]

**Supplemental Table 5.** Marginal benefits of replacing iron-folic acid tablets with multiple micronutrient tablets for pregnant women in Burkina Faso based on equal-dose iron trials: Number of cases of stillbirths, mortality, and adverse birth outcomes averted in 2018, and USD per case averted, assuming 100% coverage and current coverage (~10%), and estimated using overall marginal effects of MMS over IFA from *equal-dose iron trials* (unless otherwise indicated) and incorporating effect modification of the relationship between supplementation and the selected outcomes^1^

|  | | **Number of cases averted** | | | | **USD per case averted** | | | |
| --- | --- | --- | --- | --- | --- | --- | --- | --- | --- |
|  |  | **100% Coverage** | | **Current Coverage** | | **100% Coverage** | | **Current Coverage** | |
| **Stillbirths** | Overall effect | 1,106 | 96 | | $539.56 | | $627.22 | |  |
|  | Effect Modifier (n/a) | No EM | No EM | | No EM | | No EM | |  |
| **Early Neonatal Mortality** | Overall effect | 0 | 0 | | n/a | | n/a | |  |
|  | Effect Modifier (infant sex) | 944 | 90 | | $631.79 | | $674.98 | |  |
| **Neonatal Mortality** | Overall effect | 0 | 0 | | n/a | | n/a | |  |
|  | Effect Modifier (infant sex) | 1,600 | 152 | | $372.93 | | $398.43 | |  |
| **Infant Mortality** | Overall effect | 0 | 0 | | n/a | | n/a | |  |
|  | Effect Modifier (infant sex and presence of skilled birth attendant) | 2,968 | 281 | | $201.02 | | $214.93 | |  |
| **Low Birth Weight** | Overall effect | 10,320 | 1,050 | | $57.82 | | $57.59 | |  |
|  | Effect Modifier (maternal anemia) | 10,367 | 1,512 | | $57.55 | | $39.97 | |  |
| **Very Low Birth Weight** | Overall effect | 5,578 | 567 | | $106.97 | | $106.54 | |  |
|  | Effect Modifier (n/a) | -- | -- | | -- | | -- | |  |
| **Very Preterm Birth** | Overall effect | 2,900 | 277 | | $205.78 | | $218.04 | |  |
|  | Effect Modifier (maternal underweight) | -- | -- | | -- | | -- | |  |
| **Preterm Birth** | Overall effect | 5,846 | 595 | | $102.07 | | $101.66 | |  |
|  | Effect Modifier (maternal underweight) | 5,747 | 572 | | $103.82 | | $105.66 | |  |
| **SGA Oken** | Overall effect | 0 | 0 | | n/a | | n/a | |  |
|  | Effect Modifier (n/a) | 6,032 | 880 | | $98.93 | | $68.70 | |  |
| **SGA Intergrowth** | Overall effect | 7,805 | 794 | | $76.45 | | $76.14 | |  |
|  | Effect Modifier (n/a) | -- | -- | | -- | | -- | |  |
| **Total Mortality (Stillbirths + Infant Mortality)** | Varied (no EM for stillbirth; infant sex and presence of skilled birth attendant for infant mortality) | 4,074 | 378 | | $146.46 | | $160.08 | |  |

^1^Results assume that pregnant women who are covered each receive and consume 180 capsules per pregnancy and that tablets are imported. Effect sizes for estimation of cases averted are taken from Smith et al. (2017), using the subset of trials in which equal doses of iron were administered in the MMS and IFA groups. IFA, iron-folic acid. MMS, multiple micronutrient supplement.

^2^Data on effect modifiers for very low birth weight, very preterm birth, and for SGA assessed by the Intergrowth standard were not provided in the equal-dose trial results reported in Smith et al. (2017).
