# Supplementary material for: Replacing iron‐folic acid with multiple micronutrient supplements among pregnant women in Bangladesh and Burkina Faso: costs, impacts, and cost‐effectiveness
Source: Ann N Y Acad Sci. 2019 May 27;1444(1):35–51. doi: 10.1111/nyas.14132 (PMC6771790; doi:10.1111/nyas.14132)
Supplement: Supplementary file 6 — Supplementary Table S6. Marginal benefits of replacing iron‐folic acid tablets with multiple micronutrient capsules for pregnant women in Bangladesh based on equal‐dose iron trials and all trials: Disability‐Adjusted‐Life Years (DALYs) in 2018 and USD per DALY averted, assuming either 100% coverage and current coverage and estimated using overall marginal effects of MMS over IFA from equal‐dose iron trials (unless otherwise indicated) and incorporating effect modification of the relationship between supplementation and the selected outcomesa [file NYAS-1444-35-s006.docx]

**Supplemental Table 6.** Marginal benefits of replacing iron-folic acid tablets with multiple micronutrient capsules for pregnant women in Bangladesh based on equal-dose iron trials and all trials: Disability-Adjusted-Life Years (DALYs) in 2018 and USD per DALY averted, assuming either 100% coverage and current coverage and estimated using overall marginal effects of MMS over IFA from *equal-dose iron trials* (unless otherwise indicated) and incorporating effect modification of the relationship between supplementation and the selected outcomes^1^

|  |  | **Number of YLLs, YLDs, DALYs averted** | | **USD per YLL, YLD, DALY averted** | |
| --- | --- | --- | --- | --- | --- |
|  |  | **100% Coverage** | **Current Coverage** | **100% Coverage** | **Current Coverage** |
| **YLL (Mortality)** | Overall effect | 148,530 | 72,678 | $18.00 | $19.22 |
|  | Effect Modifier (multiple) | 358,251 | 180,241 | $7.46 | $7.75 |
| **YLD (Low Birth Weight)** | Overall effect | 234,378 | 122, 486 | $11.41 | $11.40 |
|  | Effect Modifier (maternal anemia) | 159,377 | 101,393 | $16.78 | $13.77 |
| **YLD (Preterm Birth)** | Overall effect | 90,414 | 47,250 | $29.58 | $29.56 |
|  | Effect Modifier (maternal underweight) | 100,061 | 51,811 | $26.73 | $29.96 |
| **Total (Mortality + LBW)** | Overall effect | 382,908 | 195,163 | $6.98 | $7.16 |
|  | Effect Modifier (multiple) | 517,629 | 281,634 | $5.17 | $4.96 |
| **Total (Mortality + Preterm)** | Overall effect | 238,944 | 119,928 | $11.19 | $11.65 |
|  | Effect Modifier (multiple) | 458,313 | 232,052 | $5.83 | $6.02 |

^1^Results assume that pregnant women who are covered each receive and consume 180 capsules per pregnancy and that tablets are imported. Effect sizes for estimation of cases averted are taken from Smith et al. (2017), using results from the subset of trials in which equal doses of iron were administered in the MMS and IFA groups. IFA, iron-folic acid. MMS, multiple micronutrient supplement.
