# Supplementary material for: Replacing iron‐folic acid with multiple micronutrient supplements among pregnant women in Bangladesh and Burkina Faso: costs, impacts, and cost‐effectiveness
Source: Ann N Y Acad Sci. 2019 May 27;1444(1):35–51. doi: 10.1111/nyas.14132 (PMC6771790; doi:10.1111/nyas.14132)
Supplement: Supplementary file 8 — Supplementary Table S8. Sensitivity of cost‐effectiveness of replacing iron‐folic acid tablets with multiple micronutrient tablets for pregnant women in Bangladesh: USD per DALY averted under low, best‐guess, and high marginal costs of multiple micronutrient tablets, assuming current national coverage (∼50%) and estimated using overall marginal effects of MMS over IFA from all trials and incorporating effect modification of the relationship between supplementation and the selected outcomesa [file NYAS-1444-35-s008.docx]

**Supplementary Table 8**: Sensitivity of cost-effectiveness of replacing iron-folic acid tablets with multiple micronutrient tablets for pregnant women in Bangladesh: USD per DALY averted under low, best-guess, and high marginal costs of multiple micronutrient tablets, assuming current national coverage (~50%) and estimated using overall marginal effects of MMS over IFA from all trials and incorporating effect modification of the relationship between supplementation and the selected outcomes^1^

|  |  | **Low marginal cost**  **($US 0.002439/tablet)** | **Best-guess marginal cost ($US 0.004878/tablet)** | **High marginal cost ($US 0.009757/tablet)** |
| --- | --- | --- | --- | --- |
| **YLL (Mortality)** | Overall effect | $12.01 | $24.02 | $48.04 |
|  | Effect Modifier (multiple) | $2.74 | $5.48 | $10.97 |
| **YLD (Low Birth Weight)** | Overall effect | $5.70 | $11.40 | $22.80 |
|  | Effect Modifier (maternal anemia) | $5.34 | $10.67 | $21.34 |
| **YLD (Preterm Birth)** | Overall effect | $14.78 | $29.56 | $59.11 |
|  | Effect Modifier (maternal underweight) | $14.23 | $28.46 | $56.93 |
| **Total (Mortality + LBW)** | Overall effect | $3.87 | $7.73 | $15.46 |
|  | Effect Modifier (multiple) | $1.81 | $3.62 | $7.24 |
| **Total (Mortality + Preterm)** | Overall effect | $6.63 | $13.25 | $26.50 |
|  | Effect Modifier (multiple) | $2.30 | $4.60 | $9.19 |

^1^Results assume that pregnant women who are covered each receive and consume 180 capsules per pregnancy and that tablets are imported. Effect sizes for estimation of cases averted are taken from Smith et al. (2017), using results from all included trials. DALY, disability-adjusted life year; IFA, iron-folic acid; MMS, multiple micronutrient supplement; YLD, years lived with disability; YLL, years of life lost.
